# *Ex vivo* pharmacokinetic/pharmacodynamic of hexahydrocolupulone against *Clostridium perfringens* in broiler chickens

**DOI:** 10.3389/fvets.2024.1362292

**Published:** 2024-05-02

**Authors:** Wanying Zhang, Yixing Lu, Minglang Ma, Jinyu Yang, Huiguo Huang, Xianfeng Peng, Zhenling Zeng, Dongping Zeng

**Affiliations:** ^1^Guangdong Provincial Key Laboratory of Veterinary Pharmaceutics Development and Safety Evaluation, College of Veterinary Medicine, South China Agricultural University, Guangzhou, China; ^2^National Risk Assessment Laboratory for Antimicrobial Resistance of Animal Original Bacteria, Guangzhou, China; ^3^Guangzhou Insighter Biotechnology Co., Ltd., Guangzhou, China

**Keywords:** hexahydrocolupulone, *Clostridium perfringens*, pharmacokinetic/pharmacodynamic (PK/PD), broiler, *ex vivo*

## Abstract

The economic impact of necrotizing enteritis (NE) resulting from *Clostridium perfringens* infection has been significant within the broiler industry. This study primarily investigated the antibacterial efficacy of hexahydrocolupulone against *C. perfringens*, and its pharmacokinetics within the ileal contents of broiler chickens. Additionally, a dosing regimen was developed based on the pharmacokinetic/pharmacodynamic (PK/PD) model specific to broiler chickens. Results of the study indicated that the minimum inhibitory concentration (MIC) of hexahydrocolupulone against *C. perfringens* ranged from 2 mg/L to 16 mg/L in MH broth. However, in ileal content, the MIC ranged from 8 mg/L to 64 mg/L. The mutation prevention concentration (MPC) in the culture medium was found to be 128 mg/L. After oral administration of hexahydrocolupulone at a single dosage of 10–40 mg/kg bodyweight, the peak concentration (*C*_max_), maximum concentration time (*T*_max_), and area under the concentration-time curve (AUC) in ileal content of broiler chickens were 291.42–3519.50 μg/g, 1–1.5 h, and 478.99–3121.41 μg h/g, respectively. By integrating the *in vivo* PK and *ex vivo* PD data, the AUC_0-24h_/MIC values required for achieving bacteriostatic, bactericidal, and bacterial eradication effects were determined to be 36.79, 52.67, and 62.71 h, respectively. A dosage regimen of 32.9 mg/kg at 24 h intervals for a duration of 3 days would yield therapeutic efficacy in broiler chickens against *C. perfringens*, provided that the MIC below 4 mg/L.

## 1 Introduction

The incidence of *Clostridium perfringens*-induced necrotic enteritis (NE) in the broiler industry, as well as other subclinical diseases associated with the bacteria, has increased (1, 2). Due to the significant costs associated with disease in broiler production (3), the annual cost of NE to the global poultry industry is estimated to be approximately $6 billion, including production losses and the cost of management measures (4). Antibiotic growth promoters (AGPs) can sustain intestinal well-being and modify the composition of resident microorganisms, consequently enhancing production efficiency and ameliorating intestinal health in broiler chickens (5). In recent years, numerous countries worldwide, including the European Union and the United States, have ceased the utilization of antibiotics as growth promoters in poultry feed due to the escalating resistance of *C. perfringens* to these medications (6, 7). When high concentrations of antibiotics were used, their residues can be found in the blood, the other tissues of the poultry and feces (8). The drug excreted in the feces of treated animals, and can contaminate the feed of other untreated animals (9). Vegetables may also be contaminated from feces especially in countries in where feces are generally used as a fertilizer (10). This presents a significant risk to public health, underscoring the urgency to find herbal or natural products to replace antibiotics.

**Figure 1:**
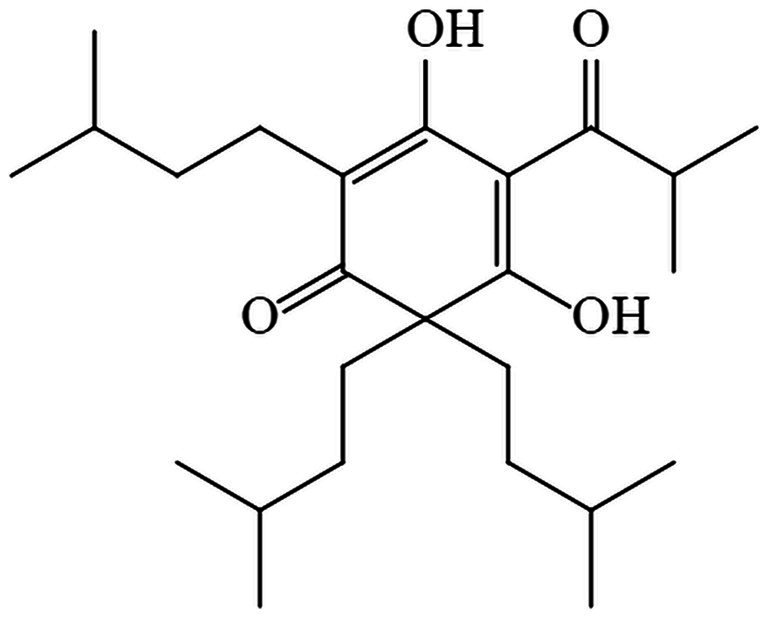
Hexahydrocolupulone structural formula.

As research progresses, certain natural antimicrobial agents utilized in food preservation have demonstrated exceptional efficacy in suppressing microbial proliferation (11). Some of the active ingredients extracted from the plant are considered safe and reliable (12). Hop *(Humulus lupulus* L.) is a dioecious vine belonging to the genus *Humulus* of the Cannabis family and widely cultivated around the world (13). Hop-derived bitter acids and their oxidation products not only give the unique bitter taste and aroma of beer but also exert a wide range of biological effects, including antibacterial (14), anti-inflammation (15), antifibrogenesis (16), and they have been considered as chemopreventive agents. β-acid, a member of bitter acids (17), contains a blend of lupulone homologs such as lupulone, colupulone, and adlupulone (13). Although β-acids have antimicrobial effects, they are unstable and are easily oxidized (18). However, as hydrogenated derivatives of β-acids, hexahydro-β-acids (HBA) are mixtures of analogues such as hexahydrolupulone, hexahydrocolupulone, and hexahydroadlupulone (19). The stability, antibacterial and antioxidant activity of HBA are better than those of β-acids (19, 20). HBA can inhibit the expression of proteins related to DNA replication, transcription, translation, and proteins related to ribosome synthesis of *Listeria monocytogenes*, resulting in a decrease in protein content in cells, thereby hindering normal life activities and physiological metabolism (21). Based on the Federal Regulations of Food and Drug Administration (FDA), hops and their extracts are widely acknowledged as safe (22). The structural formula of hexahydrocolupulone is shown in Figure 1.

The objective of this study was to investigate the pharmacokinetic (PK) and pharmacodynamic (PD) properties hexahydrocolupulone in the ileal content. The inhibitory *I*_max_ model was employed to compute the PK/PD indices necessary for varying levels of antibacterial efficacy. Moreover, the dosage regimen of hexahydrocolupulone in broiler chickens facilitated the determination of an efficacious dose for NE.

## 2 Materials and methods

### 2.1 Chemicals

Hexahydrocolupulone (98.6%) was provided by Guangzhou Insighter Biotechnology (Guangzhou, China). Mueller–Hinton (MH) broth and MH agar were obtained from Qingdao Hope Bio-Technology Co., Ltd (Qingdao, China). Tryptone–sulfite–cycloserine (TSC) agar was obtained from Guangdong Huankai Microbial Technology (Guangdong, China).

### 2.2 Bacteria

A total of 26 isolates of *C. perfringens* were employed in this study, comprising one standard strain (ATCC 13124) procured from the Chinese Veterinary Culture Collection Center and 25 strains derived from broiler chickens in Guangdong Province from 2021 to 2023. All strains were stored at −80°C until use. Before each experiment, these bacteria cultures were subcultured on TSC agar and incubated at 37°C for 18–24 h.

### 2.3 Animals

This study utilized two-week-old Sanhuang broiler chickens with a weight of 50 ± 5 g, which were in a healthy condition. Prior to conducting the experiments, the broiler chickens underwent a 7 days acclimation period. Throughout the study, the broiler chickens were provided with unrestricted access to antibiotic-free food and water. Food, but not water, was withheld for 12 h before dosing and until 4 h after drug administration. All procedures conducted in this study were approved by the Institutional Animal Care and Use Committee of South China Agricultural University, with the assigned approval number of 2022A016.

### 2.4 Determination of MIC, MBC, and MPC

The sensitivity of hexahydrocolupulone selected in MH broth was evaluated using the microdilution method recommended by CLSI (23). Following a 24 h incubation period, the MIC was established as the lowest concentration of hexahydrocolupulone that effectively hindered observable bacterial growth. Additionally, the ileal contents were assessed for MIC through the microdilution technique. To determine the minimal bactericidal concentration (MBC), 100 μL suspension from the MIC determination wells was consecutively diluted 10 fold in broth. The colony-forming unit of each dilution was counted by spreading 20 μL onto TSC agar plates after 24 h incubation at 37°C in anaerobic condition. The MBC was determined as the concentration at which a 99.9% reduction in the bacterial counts was achieved. The agar method was employed to determine the MPC of hexahydrocolupulone (24). The *C. perfringens* strains with a concentration of 10^10^ CFU/mL were inoculated to agar plates that contained varying concentrations of hexahydrocolupulone (1 MIC, 2 MIC, 4 MIC, 8 MIC, 16 MIC, and 32 MIC). These plates were then incubated at 37°C for 72 h. The MPC was defined as the concentration of hexahydrocolupulone that did not facilitate the growth of bacteria on the agar plates.

### 2.5 *In vitro* and *ex vivo* time-killing curves

Different concentrations of hexahydrocolupulone (1/4 MIC, 1/2 MIC, 1 MIC, 2 MIC, and 4 MIC) were prepared in MH broth. Test tubes were inoculated with 10^6^ CFU/mL *C. perfringens* and incubated at 37°C. Bacterial counts (CFU/mL) were determined at 0, 1, 2, 4, 8, 12, and 24 h of incubation. Specifically, 100 μL of culture was collected at each time point and serially diluted. Colony counts were performed the following morning with a limit of detection (LOD) of 50 CFU/mL. All experiments were conducted in triplicate.

The ileal contents were collected at different intervals after oral administration of 20 mg/kg hexahydrocolupulone in a PK test for high-speed centrifugation and sterile filter treatment. Subsequently, an *ex vivo* time-kill curve was established. Viable bacteria were enumerated at specific time points (0, 1, 2, 4, 8, 12, and 24 h) by incubating tubes containing the bacterial culture and intestinal contents at 37°C. The LOD for viable bacteria was set at 50 CFU/mL.

### 2.6 Pharmacokinetic of hexahydrocolupulone in broiler chickens ileum content

Following a period of 7 days of acclimatization, the 144 chickens were subjected to random allocation into three distinct groups, each comprising 48 chickens. The administered doses were 10, 20, and 40 mg/kg. Subsequently, at specific time intervals after the oral administration of hexahydrocolupulone (0.083, 0.25, 0.5, 0.75, 1, 1.5, 2, 4, 6, 8, 12, and 24 h), four broiler chickens were euthanized in each group to collect ileum contents.

The ileum content was accurately weighed to 0.2 ± 0.02 g and then added with 600 μL of 1% formic acid methanol. The mixture was vortexed for 1 min and centrifuged at 13,000 rpm for 10 min. About 200 μL of supernatant was added with 400 μL of 1% formic acid methanol. The mixture was vortexed for 1 min at 13,000 rpm, centrifuged for 10 min, passed through a 0.22 μm filter membrane, and analyzed by high-performance liquid chromatography (HPLC).

The determination of hexahydrocolupulone concentrations in intestinal contents by using HPLC and UV detectors involved the following conditions: UV detection at 341 nm, a column temperature of 30°C, a mobile phase consisting of water with 0.05% phosphoric acid and methanol, and a sample size of 20 μL injected into the HPLC system (Shimazu LC-20A) with a flow rate of 1 mL/min. Separation was achieved using an A R D-C_18_ (250 mm × 4.6 mm, 5 μm) column. The calibration range for this analysis was 0.25–20 μg/g. The precision levels for intraday and interday measurements ranged from 1.54 to 6.74% and from 4.82 to 7.43%, respectively. The LOD and limit of quantification (LOQ) were determined to be 0.10 and 0.25 μg/g, respectively. A non-compartmental analysis of hexahydrocolupulone concentrations in the intestinal content was conducted using Phoenix WinNonlin^®^ 8.4 (Certara, L.P., Princeton, NJ, United States).

### 2.7 Analysis of the PK/PD relationship

The *ex vivo* PK/PD relationships of hexahydrocolupulone in the intestine were simulated using the *I*_max_ model in WinNonlin^®^ 8.4 (Certara, L.P., Princeton, NJ, United States) with the following equation (25):

$$E=E_{0}-\frac{I_{\text{max}}\cdot X}{{IC}_{50}+X}$$

In this study, E_0_ denotes the difference in bacterial count expressed as log_10_CFU/mL in control samples. *I*_max_ is the maximum inhibition of antimicrobial growth, determined by the alteration in log_10_CFU/mL subsequent to hexahydrocolupulone treatment. *X* represents the predictive variable, specifically the ratio of area under the concentration-time curve from 0 h to 24 h to MIC (AUC_0-24h_/MIC). *IC*_50_ denotes the *X* value that elicits 50% of the maximum antibacterial effect.

The potential optimal dosage can be calculated using the following equation (26, 27):

$$\mathrm{Dose}=\frac{\left(AUC/\mathrm{MIC}\right)\cdot \mathrm{MIC}\cdot Cl}{fu\cdot F}$$

where dose (per day) is at a steady state; CL is the clearance per day; AUC/MIC is the targeted endpoint for optimal efficacy in hours; MIC is the target pathogen; F is the bioavailability factor, and *fu* is the free fraction of the drug.

## 3 Results

### 3.1 MIC, MBC, and MPC of hexahydrocolupulone against *C. perfringens*

**Figure 2:**
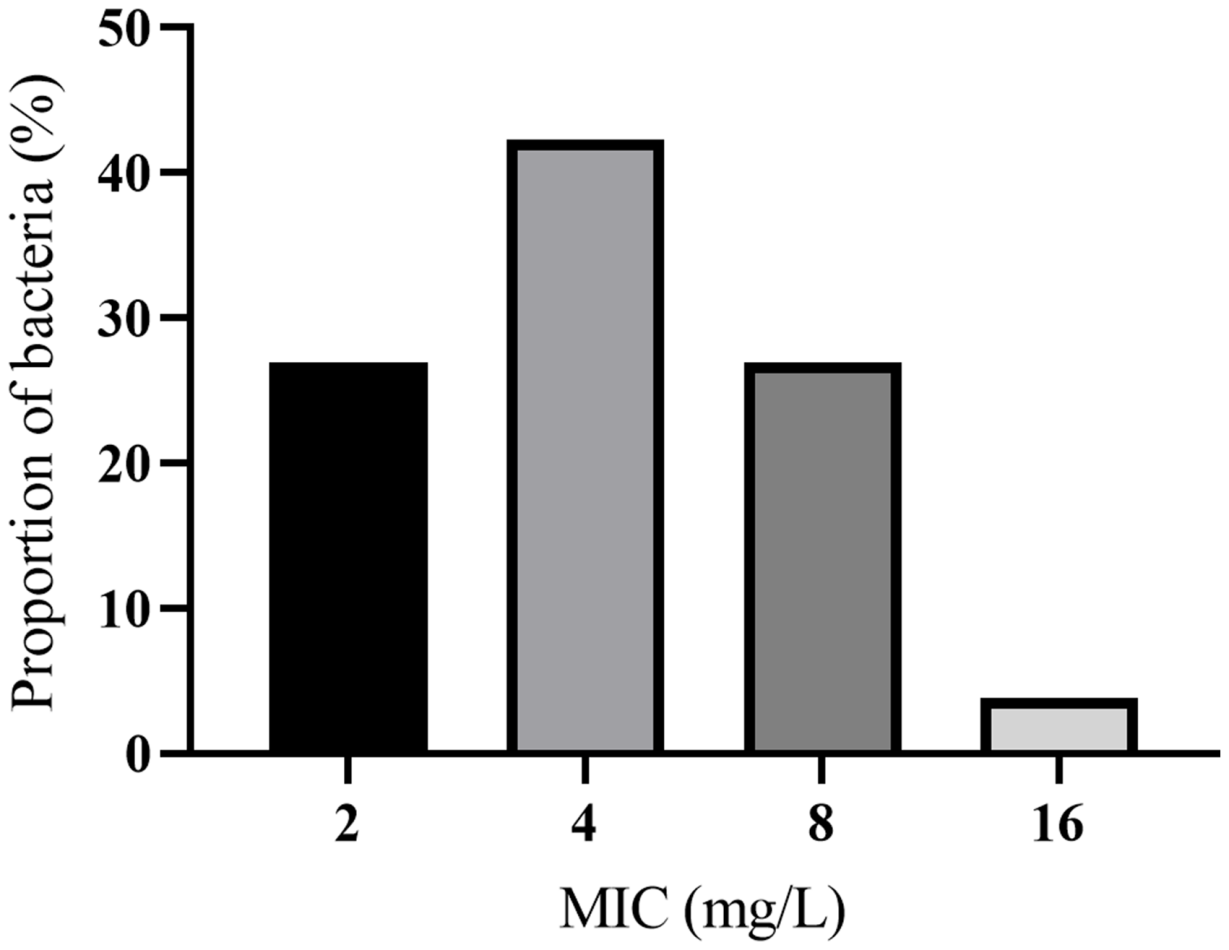
MIC distributions of hexahydrocolupulone against 26 *C. perfringens*.

A range of 2–16 mg/L was observed in the MIC of hexahydrocolupulone against 26 strains of *C. perfringens*. The percentages of each MIC (2, 4, 8, and 16 mg/L) were 26.92, 42.31, 26.92, and 3.85%, respectively. The distribution of MICs is depicted in Figure 2. In MH broth, the MIC and MBC of hexahydrocolupulone against *C. perfringens* ATCC13124 were 4 and 16 mg/L, respectively; however, in ileal content, these concentrations were sixteen times higher at 16 and 64 mg/L, respectively. The MPC in the culture medium was found to be 128 mg/L, which was 32 times the MIC. The MIC of hexahydrocolupulone against *C. perfringens* ATCC13124 in the ileum content was determined to be 16 μg/mL, as shown in Table 1.

**Table 1:** Antibacterial activity of hexahydrocolupulone against *C. perfringens* ATCC 13124.

	MIC (mg/L)	MBC (mg/L)	MPC (mg/L)
Artificial medium	4	16	128
Ileal content	16	64	–

The calculated MIC of hexahydrocolupulone for *C. perfringens* ATCC13124 in the ileum content (16 mg/L) was found to be four times higher than that in MH broth (4 mg/L), indicating a strong reinforcing effect of MH broth. To further validate the effects of MH broth, we determined the MICs of hexahydrocolupulone in MH broth and ileum content against 26 selected *C. perfringens* isolates. Interestingly, a significant difference in the geometric mean MIC values was observed between MH broth and ileal content, resulting in an ileal content/MH ratio of 5.57 for MICs (*p* < 0.01; Table 2).

**Table 2:** Attenuative effect of matrix on *in vitro* susceptibility of hexahydrocolupulone against *C. perfringens* (*n* = 26).

Test matrix^a^	MIC (Mean ± SD, mg/L)
Ileal content	26.46 ± 16.27
MH	4.75 ± 2.63
Ileal content/MH^b^	5.57

a MIC represent geometric means (SD) using 26 *C. perfringens* isolates.

b Comparison of *C. perfringens* serum/test medium (MH)/ratio differences: *p* < 0.01.

### 3.2 *In vitro* and *ex vivo* antimicrobial activity

**Figure 3:**
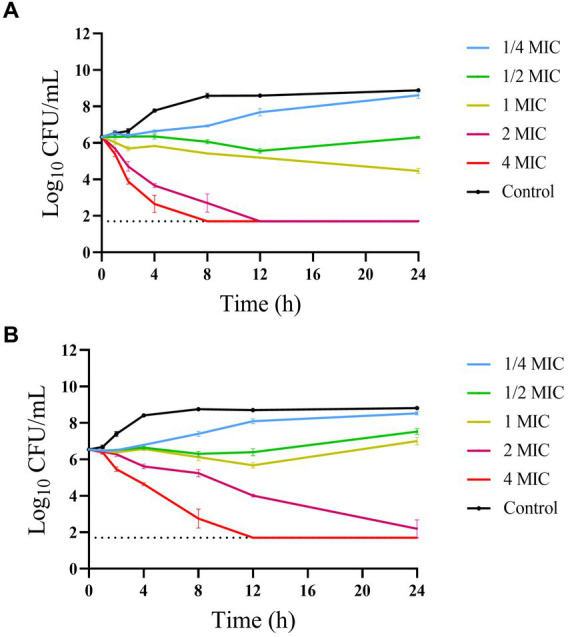
*In vitro* time-kill curve of hexahydrocolupulone against *C. perfringens* ATCC13124 **(A)** and GDZ21C61W **(B)**.

The time-kill curves of hexahydrocolupulone against *C. perfringens* ATCC13124 and GDZ21C61W in MH broth are depicted in Figure 3. The curves revealed that hexahydrocolupulone exhibited a concentration-dependent bactericidal effect. Notably, a sustained inhibitory impact on bacterial growth was observed when *C. perfringens* was exposed to hexahydrocolupulone concentrations exceeding 8 mg/L.

**Figure 4:**
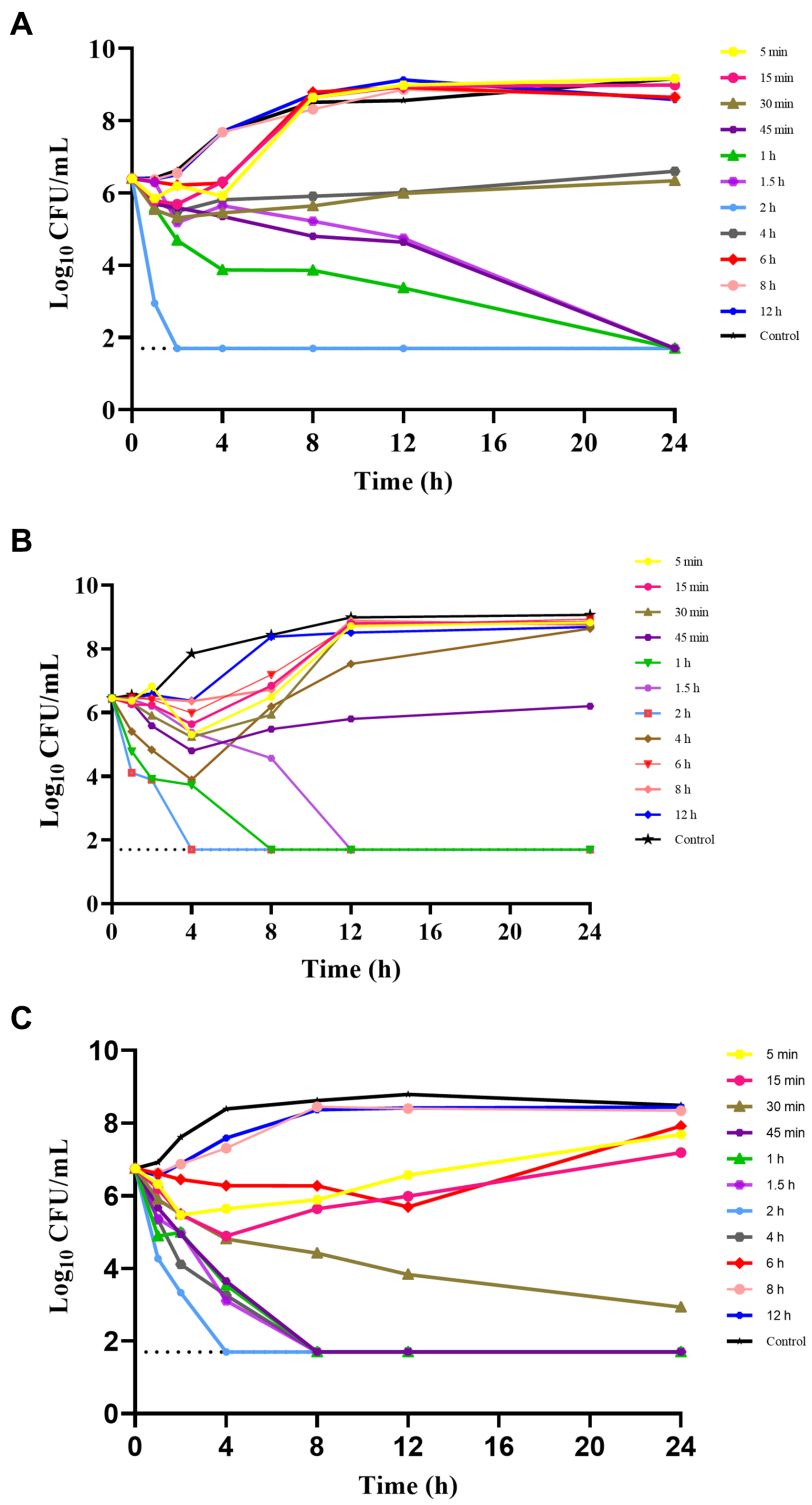
*Ex vitro* time-kill curve of hexahydrocolupulone against *C. perfringens* ATCC13124 **(A)**, GDZ21C61W **(B)**, and GDZ21C222W **(C)**.

The *ex vivo* time-kill curves were used to assess the effects of hexahydrocolupulone on samples collected at various time points. The *ex vivo* time-kill curves of hexahydrocolupulone against *C. perfringens* ATCC13124, GDZ21C61W, and GDZ21C222W are presented in Figure 4. The results indicated that hexahydrocolupulone exhibited a concentration-dependent effect in *ex vivo*, which was consistent with the observed time-kill curves *in vitro*. Notably, a significant reduction in bacterial count was observed in the high-concentration group (at 0.75–2 h), with no detectable bacteria at 24 h.

### 3.3 Pharmacokinetics analysis

**Figure 5:**
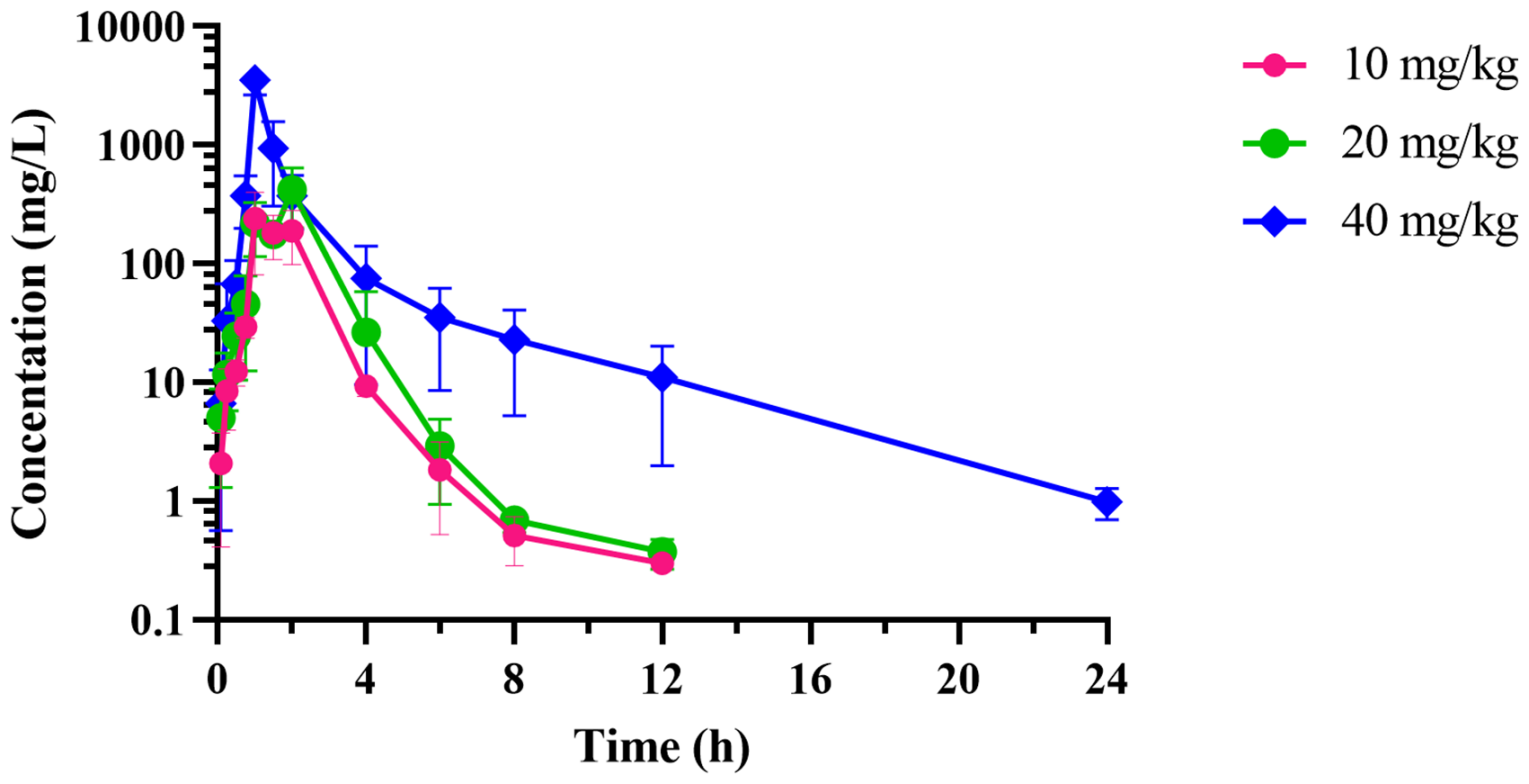
The time-concentration profile of hexahydrocolupulone in intestinal contents of broiler chickens following a single oral administration of 10, 20, and 40 mg/kg (*n* = 4).

The concentration-time curve of intestinal contents in broiler chickens after a single oral gavage at a dose of 10, 20, and 40 mg/kg was shown in Figure 5. Table 3 presents the PK parameters of hexahydrocolupulone in intestinal content. The *T*_max_ in 10 mg/kg, 20 mg/kg, and 40 mg/kg were 1.38, 1.50, and 1 h, respectively. The *C*_max_ were 291.42 ± 90.01, 440.88 ± 181.09, and 3519.50 ± 752.01 μg/g, respectively. And the AUC_last_ were 478.99 ± 149.92, 779.48 ± 210.59, and 3121.41 ± 895.08 μg h/g, respectively.

**Table 3:** Pharmacokinetic parameters of hexahydrocolupulone in ileal content following single gavage in healthy broiler chickens (*n* = 4, mean ± SD).

Dose (mg/kg)	Ileal content			
	*T*_max_ (h)	*C*_max_ (μg/g)	AUC_last_ (μg h/g)	*T*_1/2_ (h)
10	1.38	291.42	478.99	0.97
20	1.50	440.88	779.48	1.57
40	1	3519.50	3121.41	3.45

*T*_max_, time of maximum observed concentration; *C*_max_, maximum concentration; AUC_last_, the area under the concentration-time curve from 0 h to the last sample time point; *T*_1/2_, half-life.

### 3.4 PK/PD analysis

**Figure 6:**
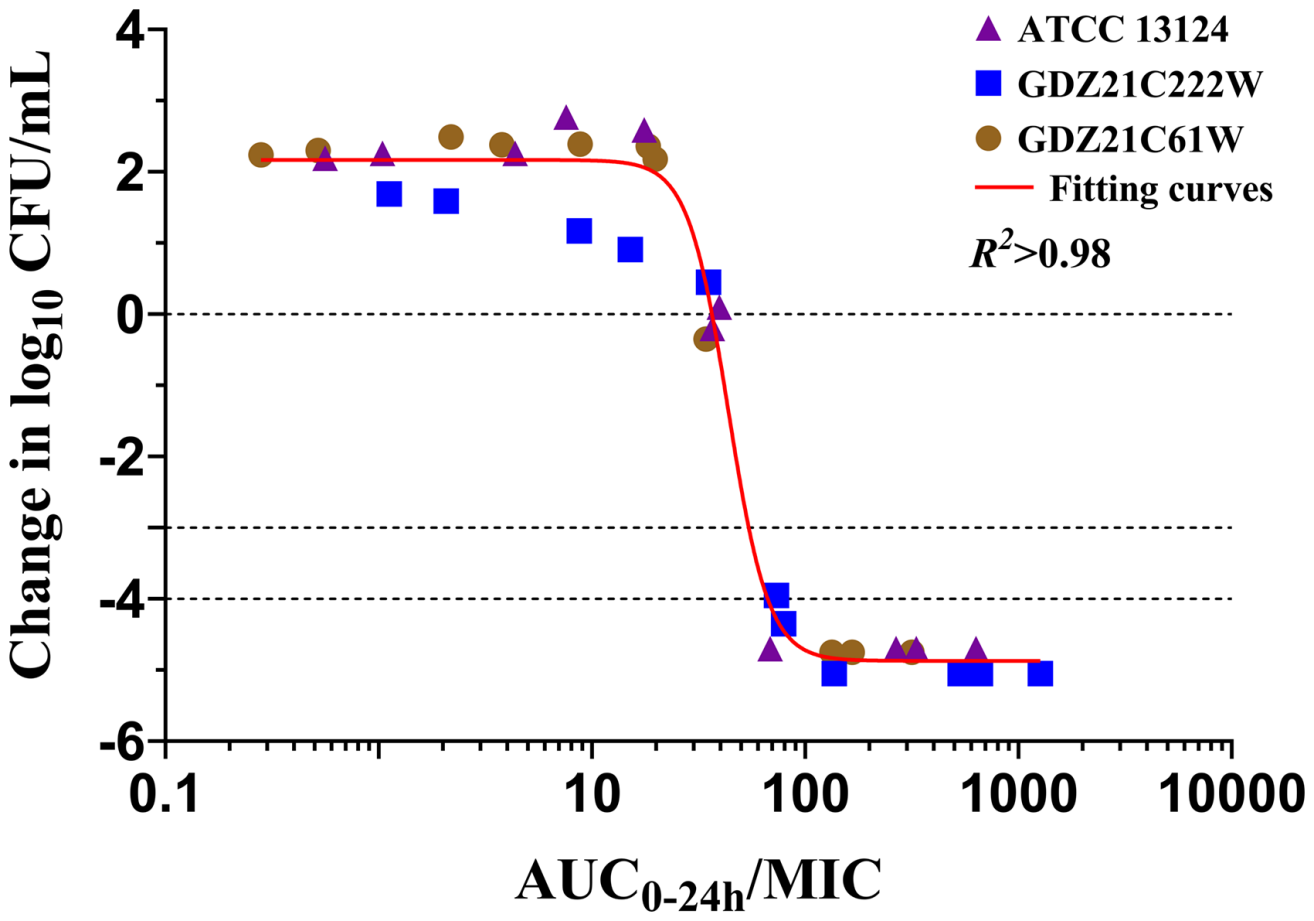
Relationships between the effect of hexahydrocolupulone against *C. perfringens* and PK/PD indices AUC_0–24h_/MIC in the *ex vivo* model. *R*^2^ is the coefficient of determination.

In ileum content, the *I*_max_ model effectively elucidated the correlation between the antimicrobial effectiveness of hexahydrocolupulone and the PK/PD parameter represented by the AUC_0-24h_/MIC ratio in the ileum. The correlation between the efficacy of hexahydrocolupulone against *C. perfringens* and each of the PK/PD indices is depicted in Figure 6. Table 4 shows the AUC_0-24h_/MIC ratios required to achieve various efficacy targets. The AUC_0-24h_/MIC values for bacteriostatic activity, bactericidal action, and virtual eradication in the ileum were 36.79, 52.67, and 62.71 h, respectively.

**Table 4:** PK/PD parameter of *ex vivo* data after oral administration hexahydrocolupulone in broiler chickens.

Parameter	Unit	PK/PD fitting parameters
*E*_0_	(log_10_CFU/mL)	2.10
*I*_max_	(log_10_CFU/mL)	6.97
IC_50_	h	43.54
AUC_0–24h_/MIC for bacteriostatic action	h	36.79
AUC_0–24h_/MIC for bactericidal action	h	52.67
AUC_0–24h_/MIC for bacterial elimination	h	62.71

*E*_0_, difference in number of bacteria counts (log_10_ CFU/mL) in a drug-free sample between 0 and 24 h; *I*_max_, difference in greatest amount of antibacterial reduction (log_10_ CFU/mL); IC_50_ is the AUC_0–24 h_/MIC value producing 50% of the maximal antibacterial effect.

## 4 Discussion

Given the increasing apprehension among consumers regarding the presence of antibiotic residues in poultry products and the emergence of antibiotic-resistant strains, substitutes for antibiotics are necessary (28, 29). Previous studies have confirmed that the active ingredients, derived or separated from hops, exhibit a substantial inhibitory effect on various pathogenic microorganisms [*Escherichia coli*, *S. aureus*, *Listeria monocytogenes* (21), and *C. perfringens* (14), and they are considered safe and reliable (12)]. The antibacterial activity of hexahydrocolupulone was evaluated using the broth microdilution method to determine the MICs of hexahydrocolupulone against 26 strains of *C. perfringens*. The MIC range of hexahydrocolupulone against clinical *C. perfringens* strains was found to be 2–32 mg/L, with more than half of the strains exhibiting MICs in the range of 2–4 μg/mL. This result indicated the strong sensitivity of *C. perfringens* to hexahydrocolupulone. This study also investigated the effect of different *ex vivo* and *in vitro* conditions, such as MH broth and ileal content, on bacterial growth and determination of MIC. The MIC of hexahydrocolupulone against *C. perfringens* ATCC13124 was found to be 4 mg/L in MH broth and 32 mg/L in the ileum. To assess the effectiveness of hexahydrocolupulone against a broad range of *C. perfringens* isolates, we determined MICs in MH broth and ileum for 26 selected isolates. The geometric means of the MICs differed significantly between MH broth and ileal content, with an ileal content/MH ratio of 5.57 for MICs. This result suggested that the ileal content has a substantial attenuative effect on the efficacy of hexahydrocolupulone. The utilization of the PK/PD index, specifically the AUC_0–24h_/MIC ratio, is highly suitable when the MIC is based on the ileal content.

On the basis of the PK findings of hexahydrocolupulone, the absorption and distribution of hexahydrocolupulone in broiler intestine were found rapid after oral administration, as evidenced by peak concentrations attained within 1.5 h; this pattern was similar to that observed with non-oral absorption medications like cyadox (30). Thus, the utilization of hexahydrocolupulone as a potential therapeutic agent against *C. perfringens* is justified, given that this bacterium predominantly targets the intestinal tract of humans and animals. AUC_last_ and C_max_ ranged from 478.88 μg h/g to 3,121.41 μg h/g and from 291.42 μg/g to 3,519.50 μg/g, respectively. This study aimed to investigate the PK data of hexahydrocolupulone in the ileum of healthy broiler chickens for PK/PD studies. Following intragastric administration, the concentrations of hexahydrocolupulone in the broiler chickens’ ileum exhibited a rapid decrease due to chyme transport, which was consistent with the PK properties observed in other orally administered non-absorbable drugs.

The approach to drug development has evolved from an empirical methodology to a modeling and simulation-based methodology, wherein the interplay between PK and PD governs the correlation between dosage and response (31). In this study, a strong correlation (*R^2^* > 0.98) was observed between the PK/PD index of AUC_0-24h_/MIC and antibacterial activity in the *ex vivo* model. The AUC_0-24h_/MIC targets necessary to achieve bacteriostatic, bactericidal, and virtual eradication effects were determined to be 36.79, 52.67, and 62.71 h, respectively. To calculate the dosage, we multiplied the MIC distribution in MH broth by a scaling factor of 5.57 to account for the differences between MH and ileal content, and the Cl/F in ileal content was measured to be 28.01 ± 9.06 mL/kg h. The use of fu was not necessary for the utilization of PD data generated in the small intestine (32). The recommended therapeutic dosage of hexahydrocolupulone for the treatment of *C. perfringens* with an MIC of ≤4 mg/L was 32.9 mg/kg, to be administered every 24 h.

Some compounds of hops have potential to be used as feed additives to broiler chickens. Some references in the literature indicate that some compounds of hops can replace antimicrobial performance enhancers in the diets of broiler chickens (33). Nevertheless, hop supplementation at the highest concentration influenced the performance of broiler chickens (34). The potency of hop as an antimicrobial agent has also been shown in poultry. The results have demonstrated that hop ß-acid lupulone supplementation to drinking water decreased caecal *C. perfringens* counts in challenged chickens in both jejunal and caecal sampling sites across all lupulone dosages tested (14). On the other hand, no significant changes were noted in the overall microbiota of the caecum or the midgut when lupulone was added to the water (35). Therefore, it is significant to investigate the PK and PD properties hexahydrocolupulone in broiler chickens.

In this study, we successfully demonstrated the efficacy of hexahydrocolupulone against *C. perfringens* through *in vitro* and *ex vivo* experiments. Additionally, we determined the AUC_0-24h_/MIC targets of hexahydrocolupulone in simulated broiler intestines. Although HBA exhibit lipophilicity, rendering them insoluble in water, which hinders homogenous dispersion (36), Lu et al. (36) successfully prepared the inclusion complex of HBA/M-β-CD so that the water solubility of HBA was enhanced by CD inclusion. These findings strongly suggested that hexahydrocolupulone holds significant promise as a novel therapeutic agent for the treatment of *C. perfringens* infection in broiler chickens.

## Data availability statement

The raw data supporting the conclusions of this article will be made available by the authors, without undue reservation.

## Ethics statement

The animal study was reviewed and approved by Institutional Animal Care and Use Committee of South China Agricultural University, with the assigned approval number of 2022A016. The study was conducted in accordance with the local legislation and institutional requirements.

## Author contributions

WZ: Writing – original draft, Writing – review & editing, Conceptualization, Data curation, Formal analysis, Methodology. YL: Conceptualization, Data curation, Formal analysis, Writing – review & editing, Investigation, Methodology. MM: Conceptualization, Data curation, Investigation, Methodology, Writing – review & editing. JY: Methodology, Writing – review & editing. HH: Methodology, Writing – review & editing. XP: Project administration, Writing – review & editing. ZZ: Funding acquisition, Project administration, Resources, Validation, Visualization, Writing – review & editing. DZ: Funding acquisition, Project administration, Resources, Validation, Visualization, Writing – review & editing.

## Supplementary material

SUPPLEMENTARY FIGURE S1 Chromatogram of a standard solution of hexahydrocolupulone.

SUPPLEMENTARY FIGURE S2 Blank ileal content chromatogram.

SUPPLEMENTARY FIGURE S3 Chromatogram of addition to the blank ileal contents with 10 μg/g of hexahydrocolupulone.

SUPPLEMENTARY FIGURE S4 Chromatogram of ileal contents sample after 1.5 h of oral administration.
