# Supplementary figures and images for: Ex vivo pharmacokinetic/pharmacodynamic of hexahydrocolupulone against Clostridium perfringens in broiler chickens
Source: Front Vet Sci. 2024 May 2;11:1362292. doi: 10.3389/fvets.2024.1362292 (PMC11097972; doi:10.3389/fvets.2024.1362292)

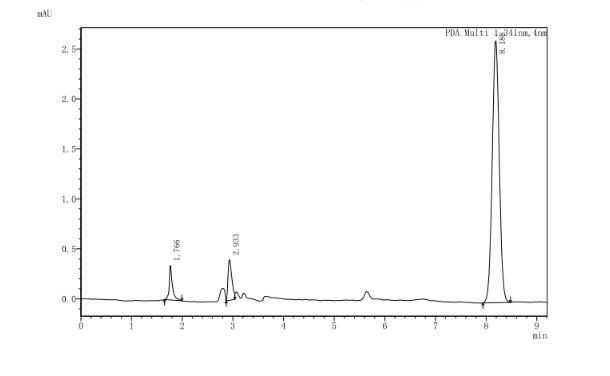

Supplement: SUPPLEMENTARY FIGURE S1 — Chromatogram of a standard solution of hexahydrocolupulone. [file Image_1.TIF]

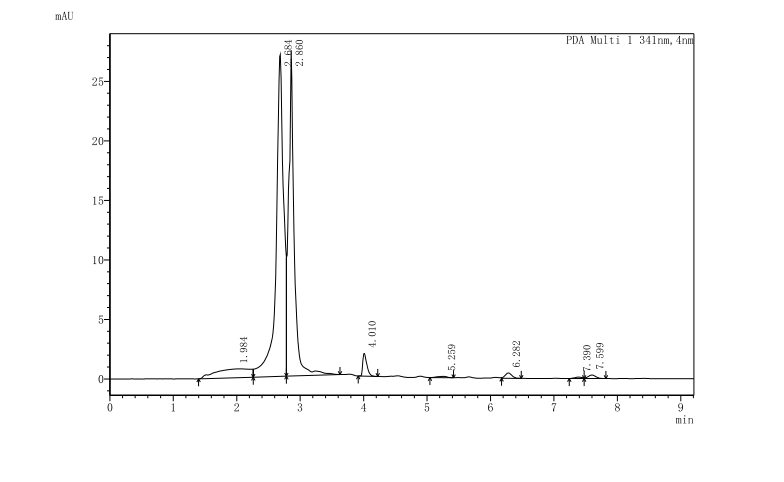

Supplement: SUPPLEMENTARY FIGURE S2 — Blank ileal content chromatogram. [file Image_2.TIF]

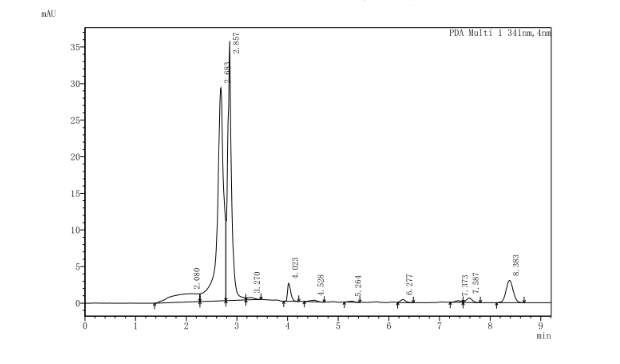

Supplement: SUPPLEMENTARY FIGURE S3 — Chromatogram of addition to the blank ileal contents with 10 μg/g of hexahydrocolupulone. [file Image_3.TIF]

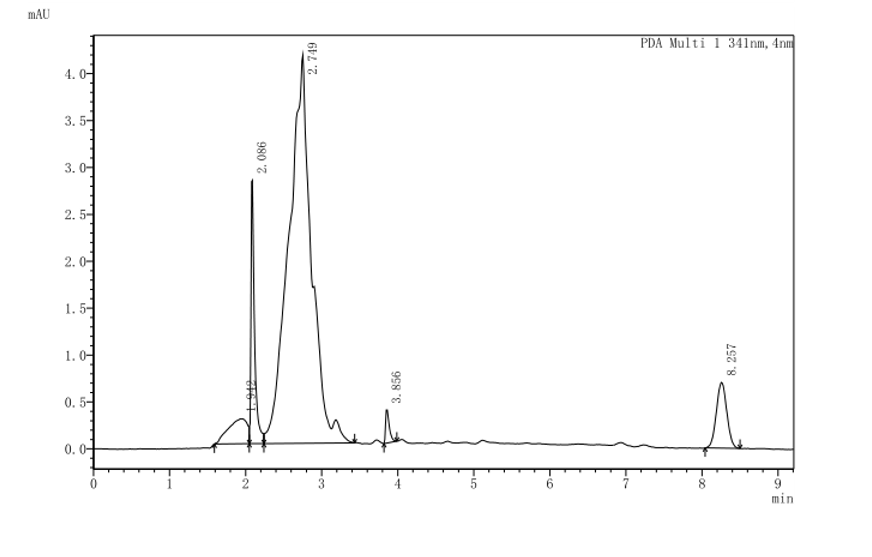

Supplement: SUPPLEMENTARY FIGURE S4 — Chromatogram of ileal contents sample after 1.5 h of oral administration. [file Image_4.TIF]
